# Traumatic Orthopaedic Injuries in the Prison Population

**DOI:** 10.5435/JAAOSGlobal-D-20-00031

**Published:** 2020-04-06

**Authors:** Daniela Barreto Rocha, Daniela Sanchez, L. Christopher Grandizio, Hemil Hasmukh Maniar, Daniel Scott Horwitz

**Affiliations:** From the Department of Orthopedics, Geisinger Medical Center, Danville, PA (Dr. Rocha, Dr. Grandizio, Dr. Maniar, Dr. Horwitz), and the Universidad del Rosario, Bogota, Colombia (Dr. Sanchez).

## Abstract

The United States has the highest population of inmates in the world (per capita and total). Musculoskeletal disorders are included within the 10 most frequent medical reports of prisoners; however, the literature about them is limited. The purpose of this study is to describe the epidemiology and complications of traumatic musculoskeletal injuries. To our knowledge, this is the first report of musculoskeletal trauma and complications in the prison population.

**Methods:**

A list of all traumatic orthopaedic injuries and complications, except for spine, was sent to the billing contractor of the Pennsylvania state prison system based on the Current Procedure Terminology, and it was queried over a 3-year period.

**Results:**

Five hundred seventy-six patients met the criteria. The hand and wrist was the most commonly injured region (65%), followed by foot and ankle (20%). Metacarpal fracture represented 22% of all injuries. A low complication rate was noted among all surgical procedures. Infection was seen in 1.15% of hand surgeries and in 2% of ankle surgeries. In addition, a low incidence of nonunion was recorded (1.5%). Nonsurgical management was the chosen method of treatment in 64% of all injuries.

**Conclusion:**

In this prison population with musculoskeletal injuries, upper extremity injuries and nonsurgical treatment are more prevalent and low energy injuries are more common. Contrary to popular belief, there is a trend toward low infection and complication rates after orthopaedic treatment. Further studies are necessary to best identify the patterns of injuries and the best way to treat inmates with orthopaedic injuries.

The United States has the highest population of inmates in the world (per capita AND total number). In 2016, the prison population was 2,121,600 corresponding with 665/100.000 prisoners/total population rate, and the level of occupancy reached 104%.^1^ Prisoners are under the supervision of regional and national governments, and these governments are required to provide healthcare services.^2^ Neglecting a prisoner's medical needs constitutes a violation to the US Constitution.^2,3^ In 2011, the state of Pennsylvania spent approximately $4705 per inmate on healthcare services. Overall US per-inmate health care spending was $6000 in the same year.^4^ Studies have reported that approximately 50% of inmates have at least one medical condition in a year, and the most prevalent pathologies were related to psychiatric disorders and infectious diseases.^5^

Musculoskeletal disorders are included within the 10 most frequent medical reports of prisoners. A study performed at a single Texas prison reported a 3% incidence of fractures.^5^ Distinctive challenges arise when providing orthopaedic care to these patients. Inmates have a propensity to present to the orthopaedic surgeon at a later stage of their pathology because many injuries or symptoms may be underreported to the prison authorities and/or due to the complexity of referral process, wherein they must be transferred outside the prison facilities escorted at all times by at least two officers. In addition, exposed implants including wires, external fixators, immobilization devices (ie, braces), or walking assistive devices may not be recommended in this population because they could be used as weapons to hurt themselves or other inmates.^3^ In addition, being in overcrowded environments can lead to poor sanitation and poor hygiene, which are unfavorable conditions for wound healing. Furthermore, the longitudinal follow-up of these patients is often difficult. For security purposes, the dates of the follow-up visits cannot be disclosed to the patient and many of these visits are not scheduled or are missed by the patients if they are transferred to other penitentiaries.^3^

Only a few studies on inmate health have been published and most focus on infectious diseases and psychiatric disorders. Although musculoskeletal diseases are frequent and comprise one of the most common health reports in prisoners, the literature about them in the inmate population is limited. Therefore, the purpose of this study is to describe the epidemiology and outcomes of musculoskeletal diseases and injuries in prisoners in the state of Pennsylvania.

## Methods

A list of all traumatic orthopaedic injuries, except for spine injuries, and complications was sent to the billing contractor of the Pennsylvania state prison system based on the Current Procedure Terminology (CPT), and it was queried over a 3-year period (September 2014 to December 2017).

Because we used only deidentified data, our study obtained an IRB exemption.

## Results

Five hundred seventy-six patients met the inclusion criteria with a total of 630 orthopaedic injuries based on the CPT codes.

Four hundred sixty-eight patients were found to have only one CPT code recorded, whereas 54 had two or more codes in the same day and 54 had variable procedures and/or codes on different dates.

Nonsurgical management was the chosen method of treatment in 64% (403/630) of all injuries.

Upper limb injuries were two and a half times more common than lower limb injuries, 72% (450) and 28% (179), respectively. One CPT code-related diagnosis was not possible to classify as upper or lower extremity injury.

Hand and wrist were the most injured regions, accounting for 65% (411/630) of all injuries and 91% of upper limb injuries (411/450). Ankle and foot injuries represented 20% (129/630) of all injuries and 72% (129/179) of lower extremity injuries. Surgical treatment was more commonly performed in three regions: pelvis and hip (16/20), ankle (49/85) and knee (12/23), whereas all others had more nonsurgical than surgical approaches. Table 1 shows the regions and treatments.

**Table 1:** Procedures by Region Based on Surgical or Nonsurgical Treatment

Region	Total (%)	Nonsurgical (%)	Surgical (%)
Hand	331 (53)	244 (74)	87 (26)
Ankle	85 (13)	36 (42)	49 (58)
Wrist	80 (13)	57 (5)	42 (5)
Foot	44 (7)	38 (86)	6 (14)
Knee	23 (4)	11 (48)	12 (52)
Forearm	21 (3)	11 (52)	10 (48)
Pelvis and hip	20 (3)	4 (20)	16 (80)
Humerus	14 (2)	7 (50)	7 (50)
Leg	7 (1)	2 (29)	5 (71)
Elbow	4 (1)	4 (100)	0 (0)
External fixator	1 (0)	0 (0)	1 (100)
Total	630 (100)	403 (64)	227 (36)

Metacarpal fractures were the single most prevalent injury in this population (22%), and nonsurgical management was the preferred modality of treatment, with 99 out 141 patients treated nonsurgically. Table 2 shows the most prevalent injuries.

**Table 2:** Most Prevalent Injuries

Injury	n	% Study
Metacarpal fracture	141	22
IP dislocation	63	10
P/M phalange fracture	61	10
Ankle fracture	56	9
Distal radius fracture	52	8
Distal phalange fracture	33	5
Metatarsal fracture	30	5
Distal fibula fracture	27	4
Scaphoid fracture	13	2
Humeral shaft fracture	12	2
MCP/IP articular fracture	12	2
Tibial plateau fracture	12	2
Proximal femur fracture	10	2
CMC 2–5 dislocation	7	1
MCP dislocation	7	1
Proximal ulna fracture	7	1
Other	87	14
Total	630	100

Of 227 surgical procedures, the most common were ankle, metacarpal, and distal radius fractures. These three corresponded to 50% of all surgically treated injuries and 18% of all injuries (Table 3).

**Table 3:** Surgically Treated Injuries

Ankle fracture	49
Metacarpal fracture	42
Distal radius fracture	24
P/M phalange fracture	19
Proximal femur fracture	10
Tibial plateau fracture	8
MCP/IP articular fracture	7
Humeral shaft fracture	6
Flexor tendon	6
Scaphoid fracture	6
Distal phalange fracture	5
Tibial shaft fracture	5
Bennett	4
Patella	4
Other	32
Total	227

Regarding nonsurgical management, the most common injuries were metacarpal fractures, interphalangeal dislocation, and phalangeal shaft fractures. These three corresponded to 50% of all nonsurgically managed injuries and 32% of all injuries (Table 4).

**Table 4:** Nonsurgical Treated Injuries

Metacarpal fracture	99
IP dislocation	60
P/M phalange fracture	42
Metatarsal fracture	28
Distal phalange fracture	28
Distal radius fracture	28
Distal fibula	27
Ankle fracture	7
MCP dislocation	7
Scaphoid fracture	7
Other	70
Total	403

Complication-related CPT codes were found in 25 patients after their initial treatment. However, we could only link the primary procedure to the complication code in 14 patients. The complications were nonunion, infection, cephalomedullary nail converted to total hip arthroplasty and compartment syndrome. We presumed that the 11 patients without an identifiable primary code were either treated initially outside the prison system or preceding the period studied.

The overall nonunion rate was 1.5%, including only the fractures' surgical and nonsurgical management. Infection occurred in 1.8% of the surgically treated injuries. Of all ankle fractures, two had fibula nonunion and one had infection after surgical treatment. Of all scaphoid fractures surgically treated, 3 (50%) had nonunion. One proximal femur fracture treated with a cephalomedullary nail was converted to total hip arthroplasty for unclear reasons. One patient with multiple injuries (upper and lower extremity) had thigh fasciotomy (compartment syndrome). See Table 5 for a list of complications.

**Table 5:** Complications After Orthopaedic Treatment

Complication	n	% Injuries	
Infection	4	1.8%	4/227
Hand	1	1.1%	
Lower limb	1	1.1%	
Patella	1	25%	
Ankle	1	2%	
Nonunion	8	1.5%	8/523
Scaphoid	5	38.5%	5/13
After surgery	3	50%	3/6
Closed	1	14%	1/7
Missed	1	7.7%	1/13
Ankle	2		
Fibula	2	4.2%	2/48
Radius/Ulna	1	10%	1/11
Compartment syndrome	1	NA	
Thigh	1		
TFN to THA	1	10%	
Total	14	2.2%	14/630

Infection rate was calculated based on all surgical procedures (227). Nonunion rate was calculated based on all fractures, excluding dislocations and tendon injuries (523). Scaphoid nonunion rate was calculated based on all scaphoid fractures or over the surgically or nonsurgical managed, when it applies. Ankle nonunion was calculated based on all ankle fractures (excluding one dislocation; 48) because the two fibula nonunion occurred after surgical treatment of ankle fractures. Radius/ulna nonunion was calculated based on all radius and/or ulna forearm fractures^11^ because the CPT code was not specific. TFN to THA rate was calculated based on all proximal femur fractures.^10^

## Discussion

Studies in the prison population are challenging, and only a few articles regarding health care in inmates have been published. Even fewer studies have addressed orthopaedic care among prisoners and none, to our knowledge, have discussed the epidemiology of traumatic orthopaedic injuries and their complications.

Of 576 patients, 468 were billed just one time and using only one CPT code. This way, we could be more confident in identifying their injuries, and we can also conclude that at least 81% of this population had isolated injuries. Similar to our finding, Zura et al^6^ showed in their epidemiologic study that 82.9% of the patients had an isolated fracture.

Fractures of the distal radius and ulna, proximal femur, and metacarpals are the most common, representing 16%, 11.7%, and 10.6% of all fractures in the general population.^7^ This prisoner population showed a different pattern, and the most common injuries were metacarpal fracture (22%), IP dislocation (10%), and phalangeal shaft fracture (10%). Hand and wrist were by far the most injured region, with 65% (411/630) of all injuries, followed by foot and ankle with 20% (129/630). Metacarpal fracture was twice as prevalent in our study as in general population. Ankle fractures corresponded with 13% of prisoners' injuries, whereas in the general population, it is estimated to be 9% of all fractures.^7^ This may be an indicator of the circumstances under which the injuries occur, such as a prison fight. In addition, the prison environment is known for a high prevalence of mental illnesses and a history of substance abuse,^8^ which may help explain fights and the resultant injuries.

Conversely, metatarsal fractures were found in 5% in our study, less than in the general population, where it is estimated to correspond to 7% of all fractures.^7^ In addition, one of the most prevalent injuries was phalangeal fracture (15%; general population 10%).^7^ In both cases, the percentages are close, and although we do not have statistical power to find a true difference, we would expect a bigger prevalence of these injuries among the prisoners, given the data relating to metacarpal fractures. The lower numbers could be due to missed diagnosis, as shown in the study by Shafic et al, where hand injuries such as these were found to be reported later and no treatment was required at that time.^3^ We could think of metatarsal injuries in the same way.

A recent epidemiological study on ankle and foot fractures^9^ showed that ankle and metatarsals are the most common fractures, with 55.7% and 12.5% of all foot and ankle fractures, respectively. In our analysis, ankle and metatarsal fractures were also the most common injuries in foot and ankle segment, with 66% and 23% of these injuries.

Nonsurgical treatment was more common in this population when compared with the rates reported in the general population. Several factors may play a role in the management decisions. Orthopaedic surgeons must balance their choice based on multiple factors, which is, compliance with treatment, fixation options, follow-up perspective, and the time of diagnosis. The best treatment course may be different than what would be for the general population if one takes into account the prison environment. Splints and orthotic devices could be used as weapons and may not be the safest option.^3^

Dy et al^10^ demonstrated that scaphoid fractures were managed nonsurgically in 71% of the cases. In our study, we found a prevalence of 2% (13/603) of scaphoid fractures, with almost 50% treated surgically. Although the numbers are small, it raises a question about the delay in presentation to medical treatment and further displacement may have led to the increase in surgical treatment.

The overall complication rate was low in the prison population that we studied. A low incidence of infection after orthopaedic surgical procedures (1.8%) contrasts with the commonly held beliefs about hygiene, environmental conditions, and care in prison. Perhaps, these low numbers can be explained by this patient population that lives in a controlled environment with limited contact to the external world, where you have limited options and activities; the opportunity for noncompliance and risky behavior is minimal. The definition of infection after fracture care varies widely in the literature, from 1% to 30%^7^ based on injury mechanism, open fractures, and comorbidities, and it is difficult to estimate its true incidence.^11^ In the prison population we studied, we found a rate of infection of 1.8%, which is at the lower end of the reported infection incidence in orthopaedic injuries. In four patella fractures that were surgically treated, one (25%) developed infection. Hand and ankle injuries had an infection rate of 1% and 2%, respectively, after surgery.

Nonunion also had a low overall rate among all the 523 fractures (1.5%). In a recent report, the nonunion rate in 18 different human bones evaluated was 4.9%.^6^ Dy et al^10^ published scaphoid nonunion rates of 10.8% and 3% after surgery and casting, respectively. However, in this prison population, scaphoid nonunion accounted for 50% of the surgically treated scaphoid fractures. This can probably be explained by a lack of immobilization postsurgically because prisoners may not have accessible help with performance of their activities of daily living. Ankle nonunion after fracture surgery occurs in less than 2% in the general population,^12^ and we found it to occur in 4% (2 fibula nonunion) among prisoners after ankle fracture surgery. Even with the numbers being small, it raises the question whether certain injuries should be immobilized for a longer period or have different protocols of treatment compared with the general population because compliance with the standard treatment protocol may not be as adequate as expected.

The other two complications in this study, cephalomedullary nail to total hip arthroplasty conversion and compartment syndrome, can be difficult to analyze because of the low incidence. The patient with compartment syndrome had multiple injuries, consistent with a high energy mechanism, although exact details are unknown. The patient with cephalomedullary nail conversion had a proximal femur fracture procedure and 2 years later underwent total hip arthroplasty. This corresponds to 10% of all proximal femur fractures, but it is impossible to make any conclusions, given the small number of cases.

Our study has several limitations. We looked at the billing records for all Pennsylvania state prisons but did not have access to basic demographic information such as social, medical, and occupational history or the time frame of injury presentations. We also had no access to other factors that are known to play a role in nonunion, such as tobacco use or comorbidities. The treatment protocols were unknown, and we do not know about compliance and completion of follow-up care. We recognize that we may have missed some data in that multiple CPT codes and/or different billing dates were found in 108 patients. Despite the limitations, our goal is to provide insight regarding traumatic orthopaedic injuries and complications in prisoners. We believe that the CPT codes provided by the billing department are enough to give us this initial assessment. These data will be important for further future studies focusing on this patient population.

## Conclusion

In a prison population with musculoskeletal injuries, upper extremity injuries and nonsurgical treatment are more prevalent and low energy injuries are more common. Contrary to popular belief, there is a trend toward low infection and complication rates after orthopaedic treatment. Further studies are necessary to better identify patterns of orthopaedic injuries in inmates and the treatment options.
